# Role of Endoscopic Ultrasound in the Diagnosis of Pancreatic Neuroendocrine Neoplasms

**DOI:** 10.3390/diagnostics11020316

**Published:** 2021-02-15

**Authors:** Tatsuya Ishii, Akio Katanuma, Haruka Toyonaga, Koki Chikugo, Hiroshi Nasuno, Toshifumi Kin, Tsuyoshi Hayashi, Kuniyuki Takahashi

**Affiliations:** Center for Gastroenterology, Teine Keijinkai Hospital, Sapporo 006-0811, Hokkaido, Japan; akatanuma@gmail.com (A.K.); toyonaga.pc@gmail.com (H.T.); kochikugo@gmail.com (K.C.); h.nasuno.sapmed1st@gmail.com (H.N.); kin_toshifumi@yahoo.co.jp (T.K.); thayashi244@gmail.com (T.H.); tkuni318@gmail.com (K.T.)

**Keywords:** endoscopic ultrasound, pancreatic tumor, pancreatic neuroendocrine neoplasms

## Abstract

Although pancreatic neuroendocrine neoplasms (PNENs) are relatively rare tumors, their number is increasing with advances in diagnostic imaging modalities. Even small lesions that are difficult to detect using computed tomography or magnetic resonance imaging can now be detected with endoscopic ultrasound (EUS). Contrast-enhanced EUS is useful, and not only diagnosis but also malignancy detection has become possible by evaluating the vascularity of tumors. Pathological diagnosis using EUS with fine-needle aspiration (EUS-FNA) is useful when diagnostic imaging is difficult. EUS-FNA can also be used to evaluate the grade of malignancy. Pooling the data of the studies that compared the PNENs grading between EUS-FNA samples and surgical specimens showed a concordance rate of 77.5% (κ-statistic = 0.65, 95% confidence interval = 0.59–0.71, *p* < 0.01). Stratified analysis for small tumor size (2 cm) showed that the concordance rate was 84.5% and the kappa correlation index was 0.59 (95% confidence interval = 0.43–0.74, *p* < 0.01). The evolution of ultrasound imaging technologies such as contrast-enhanced and elastography and the artificial intelligence that analyzes them, the evolution of needles, and genetic analysis, will further develop the diagnosis and treatment of PNENs in the future.

## 1. Introduction

Pancreatic neuroendocrine neoplasms (PNENs) are relatively rare tumors that account for 2–3% of all pancreatic tumors. However, the number of reported cases has been increasing, mainly because of the advances in various diagnostic imaging modalities. Among them, endoscopic ultrasound (EUS) has a superior sensitivity for detecting PNENs compared with computed tomography (CT) and magnetic resonance imaging (MRI). With its high resolution, and when performed by experienced hands, EUS can detect focal lesions as small as 2–5 mm [1]. Tissue acquisition using EUS with fine-needle aspiration (EUS-FNA) is essential for the diagnostic and treatment decisions. Here, we review the current literature regarding the role of EUS in the diagnosis of PNENs. Since this study focuses on diagnosis, it does not include interventional EUS, such as EUS-ablation.

## 2. Types of EUS

EUS, in which the tip of the endoscope contains a high-frequency transducer, provides high-resolution images of the pancreaticobiliary region. There are two types of scope: radial scanning (RS) and curved linear array (CL). The wide 360° scanning range of an RS scope makes it easy to grasp relationships with surrounding organs and blood vessels. It is comparatively easier to visualize imaging in affigurelignment with the organ axis [2]. By contrast, the scanning range of the CL scope is narrow (180°), and it is difficult to align with the organ axis. However, a study reported the superiority of the overall imaging capability of the CL scope compared with the RS scope for the pancreaticobiliary region [3]. The CL scope was superior in delineating the pancreatic head–body transition region, the area from the hepatic portal region to the superior bile duct, and the vascular bifurcation. The RS scope was superior in delineating the major duodenal papilla and the long axis of the bile duct/gallbladder. In addition, the CL scope can be used to collect tissue samples. In a pancreatic neuroendocrine neoplasm (PNEN) examination, the CL scope may be better for diagnostic imaging for tissue collection. The RS scope can visualize the long axis of the organ, and images similar to abdominal ultrasound, CT, and MRI can be obtained; therefore, it is necessary to use them on a case-by-case basis.

## 3. EUS for Detecting PNENs

**Figure 1:**
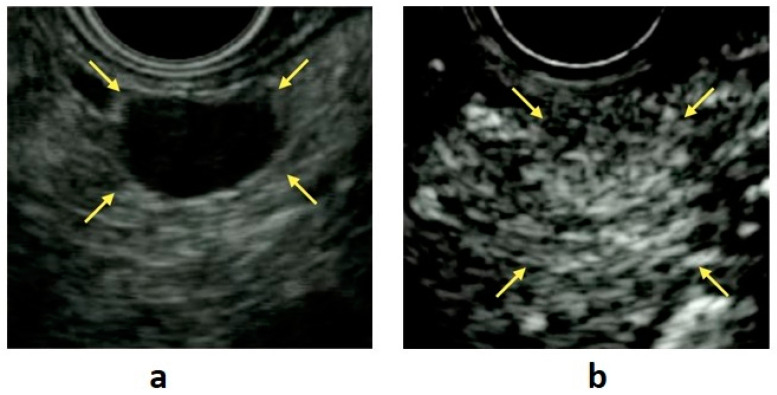
(**a**) B-mode endoscopic ultrasound (EUS): a circular hypoechoic mass is seen in the body of the pancreas (yellow arrow). (**b**) Contrast-enhanced EUS: the mass shows early enhancement compared with the surrounding pancreatic parenchyma (yellow arrow).

EUS enables detailed observation of the entire pancreas with high tissue resolution, without being affected by the gastrointestinal tract or subcutaneous fat (Figure 1a). In a systematic review, Puli et al. reported that EUS had a sensitivity of 87.2% and a specificity of 98.0% when used for the detection of PNENs [4]. Manta et al. reported that CT failed to detect 68.4% of PNENs < 10 mm and 15% of PNENs ≤ 20 mm in diameter [5]. Moreover, it has been reported that the sensitivity of CT is reduced for small lesions < 1 cm and that 91% of PNENs that are difficult to detect using multidetector-row CT can be detected with EUS [6]. James et al. reported in a meta-analysis that preoperative EUS consistently increased the overall PNEN detection rate by >25% after a CT scan, with or without additional investigative modalities such as MRI or ultrasound [7]. Thus, EUS is an essential modality for the detection of small PNENs.

The role of EUS surveillance has been controversial in recent years. PNENs can occur sporadically or as part of the hereditary multiple tumor syndromes: von Hippel-Lindau (VHL) disease and multiple endocrine neoplasia type 1 (MEN1). MEN1 or VHL patients with a PNEN may undergo imaging of the pancreas every 6 to 12 months to assess the growth rate of the tumor. Concerning the radiation risk in younger patients, EUS and MRI are preferred. In patients with MEN1, the superiority of EUS has been reported for the detection of PNENs [8,9,10,11]. However, Daskalakis et al. reported that MRI is better than EUS for the detection and subsequent surveillance of MEN1-related PNENs larger than 10 mm and it seems to be cost-effective [9]. Kappelle et al. showed that small PNENs in patients with MEN1 grow more slowly than previously suggested. The necessity of EUS surveillance for MEN1 patients with only small asymptomatic PNENs may be reduced [12].

## 4. Precautions for EUS in Functional PNENs

Nonfunctional PNENs are often asymptomatic and undetected until the tumors have grown large enough to cause a mass effect or until they metastasize. However, because functioning PNENs secrete hormones that lead to symptoms, their presence is suspected earlier, and a diagnosis is often made when the lesions are small. Functioning tumors comprise 34.5% of all PNENs [13]. Insulinomas are the most common functional tumors (20.9%), followed by gastrinomas (8.2%), glucagonomas (3.2%), vasoactive intestinal peptide-producing tumors (0.6%), and somatostatinomas (0.3%). Insulinomas tend to be smaller than other functioning PNENs because of the dramatic clinical syndrome caused by insulin secretion. Insulinomas are usually small at diagnosis, with 90% of tumors < 2 cm in diameter and 40% < 1 cm [14]. Most insulinomas occur in the pancreas, and these tumors are evenly distributed throughout the pancreatic head, body, and tail [15]. Approximately 2–10% of patients with insulinomas have multiple tumors, particularly those with MEN1 [14,16]. Thus, it is important to evaluate the entire pancreas in detail when observing insulinomas with EUS. Although insulinomas are usually sporadic, they account for 10–30% of the functioning PNENs in patients with MEN1. MEN1 coexists in 4–5% of insulinomas [15].

Gastrinomas often arise in the gastrinoma triangle, an area bounded by the junctions of the cystic duct and the common bile duct superiorly, the second and third portions of the duodenum inferiorly, and the neck and body of the pancreas medially [14]. They are more common in the duodenum than in the pancreas. Approximately 80% of sporadic lesions and 90% of lesions associated with MEN1 originate from the duodenum. Previously, the pancreas was believed to be the most common location; however, many of these masses may have been peripancreatic nodal metastases from small duodenal tumors. Pancreatic gastrinomas have an average diameter of 3–4 cm, and most are located in the pancreatic head. Duodenal gastrinomas are usually < 1 cm in diameter and are often multicentric, especially in patients with MEN1 [14]. Under EUS, it is necessary to carefully observe not only the pancreatic head but also the duodenal wall. Although most gastrinomas sporadically arise, they are the most common functioning PNENs in patients with MEN1 (20–25% of all gastrinomas occur in these patients). 

**Figure 2:**
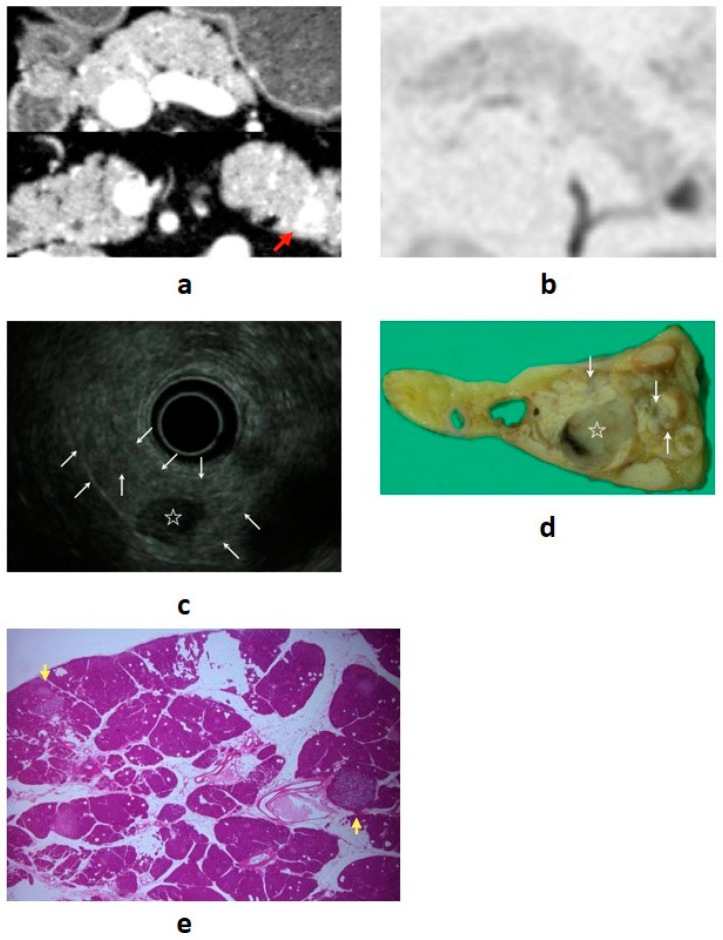
(**a**) Contrast-enhanced computed tomography (CT): a mass lesion with early hyperenhancement is seen in the tail of the pancreas (red arrow), but there are no other obvious lesions. (**b**) Diffusion-weighted magnetic resonance imaging (MRI): the mass in the pancreatic tail shows reduced diffusion. (**c**) Endoscopic ultrasound (EUS): in addition to the mass (☆) revealed by CT/MRI, many small hypoechoic masses are observed (white arrow). (**d**,**e**) Resected specimen: the main lesion (☆) is an 11 mm neuroendocrine neoplasm (NEN) G1, but multiple tumors with diameters of 1–3 mm are observed in the surrounding pancreas (white and yellow arrow) (20×). They are multiple NENs associated with multiple endocrine neoplasia type 1 (MEN-1).

As mentioned above, because PNENs and MEN1 are related, we must not forget to check for the coexistence of MEN1 in PNEN patients. Examples of multiple NENs associated with MEN-1 from our department are shown in Figure 2.

## 5. Role of EUS Elastography

Elastography measures the stiffness of the target lesion. Various types of elastography devices utilize different mechanisms. In the clinical setting, strain and shear wave elastography are mainly used for pancreatic diseases. The evaluation methods of strain elastography are classified into color pattern diagnosis, strain ratio (SR), and histogram analysis. Most of the reports so far have been on strain elastography, and there are still few reports on shear wave elastography [17]. The elastography pattern of PNENs is described as a homogeneous blue [18]. When comparing the strain ratio of a mass over the normal surrounding pancreatic parenchyma, malignant pancreatic masses and neuroendocrine tumors produce higher SR than inflammatory masses and normal parenchyma [19,20]. In one prospective study including six patients with a PNEN, the SR for PNENs was 56.73, higher than the SR of 17.41 seen in pancreatic adenocarcinoma [21]. In one study including malignant and benign PNENs, 67% sensitivity and 71% specificity were observed, with a malignant tumor SR cutoff of 4.4 [22]. However, reports on PNENs are still limited, and there are some problems in elastography in the diagnosis of pancreatic tumors [17]. Color pattern diagnosis is a qualitative method that depends on the operator. The cut-off values for each method and the definition of malignant/benign tumors for SR were different in each article. Shear wave elastography may overcome these limitations related to reproducibility and intra- and inter-observer variability because the hardness of the target is measured without a comparison area.

## 6. Role of Contrast-Enhanced EUS (CE-EUS)

CE-EUS is useful for the evaluation of pancreatic disease because it permits the observation of the hemodynamics of masses in real time. This technique is based on the fact that microbubbles in contrast agents are disrupted by ultrasound waves, producing signals that are detected by the ultrasound imager. Because typical PNENs have abundant blood vessels, these tumors show hypervascular contrast in the early phase, persisting until the delayed phase (Figure 1b). CE-EUS has a high sensitivity (78.9–95.1%) and a high specificity (98.7%) in the identification of PNENs [23,24].

Ishikawa et al. reported that heterogeneous ultrasonographic texture indicates a malignant disease [23]. Furthermore, Palazzo et al. reported that contrast-enhanced harmonic EUS (CH-EUS) can accurately predict aggressive tumor behavior by evaluating the heterogeneous patterns of PNENs, with a sensitivity of 86% and a specificity of 96% [25]. Takada et al. reported that CH-EUS with time-intensity curve analysis is useful for PNEN diagnosis and grading [26].

CE-EUS plays an important role in finding a specific site within a lesion that would be more suitable for EUS-FNA. Identification of hypervascular sites in such lesions may help avoid sampling rich fibrous areas [27].

## 7. Artificial Intelligence Analysis for Endoscopic Ultrasonography

There are reports using artificial intelligence (AI) for the EUS diagnosis of pancreatic tumors. As per a recent review, there have been seven reports so far, and the diagnostic abilities in these reports were approximately 85–95% [28]. However, only one study included PNENs [29]. CE-EUS and elastography have limitations related to reproducibility and intra- and inter-observer variability, but AI may be able to overcome these problems [30]. When using AI for the diagnosis of diseases, many images are required. Pancreatic tumors such as PNENs, acinar cell carcinoma, and mucinous cystic neoplasms are rarer than pancreatic ductal carcinoma and IPMN. Many EUS images of rare diseases are required to train AI to learn EUS diagnosis, but it is difficult to collect these images. However, AI will be an essential technique to help doctors to improve on their diagnostic ability using endoscopy and EUS in the near future.

## 8. Features of EUS Findings in PNENs

On EUS examination, PNENs typically appear as well-rounded, hypoechoic lesions with a homogeneous pattern and clear regular margins (Figure 1a,b). However, because PNENs grow expansively, they may cause cystic degeneration and calcification as their size increases. In these cases, PNENs often presented a heterogeneous pattern (Table 1).

### 8.1. Cystic Degeneration

**Figure 3:**
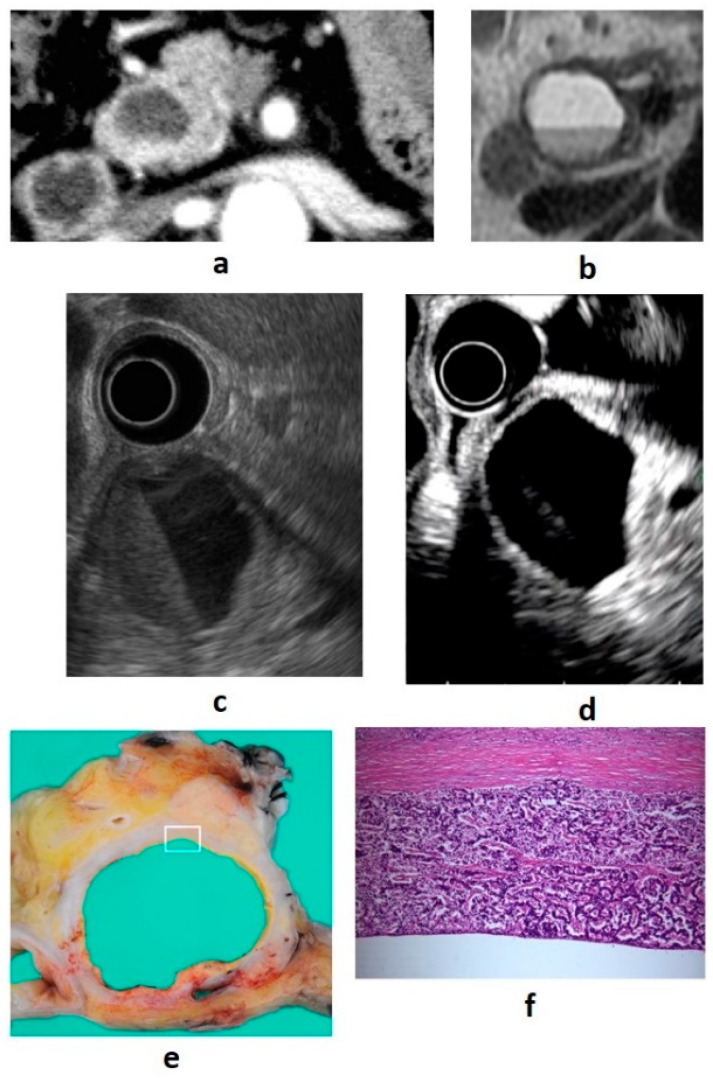
(**a**) Contrast-enhanced computed tomography (CT): a cystic lesion is seen on the pancreatic head. (**b**) Magnetic resonance imaging: fluid–fluid level formation is shown. (**c**) B-mode endoscopic ultrasound (EUS): a cystic lesion with fluid–fluid level formation and a thickened wall is seen. (**d**) Contrast-enhanced EUS: the wall is hyperenhanced compared with the surrounding pancreatic parenchyma. (**e**,**f**) Resected specimen: neuroendocrine neoplasm G1 (100×).

The most common cause of cystic degeneration is tumor bleeding, whereas tumor necrosis is rarely the cause. Cystic degeneration is mostly observed in well-differentiated PNENs. The frequency is 10–17% of all PNENs, and the larger the tumor, the higher the rate of cystic degeneration [31]. Gaujoux et al. found no association between cystic degeneration and tumor malignancy [31]. The biological behavior of cystic PNENs is somewhat less aggressive than that of solid PNENs [32,33]. Cystic degeneration is visualized as a low absorption area on contrast CT and is recognized as a nonechoic area on B-mode EUS and as an avascular area on CE-EUS. If the cysts become larger, the imaging findings will be similar to those of serous cystic neoplasms, and differentiation is necessary [34]. In EUS diagnosis, it is important to identify the solid tumor part of the cyst margin as a wall thickening or protrusion. Examples of cystic PNEN images from our department are shown in Figure 3. 

### 8.2. Pancreatic Duct Stricture

**Figure 4:**
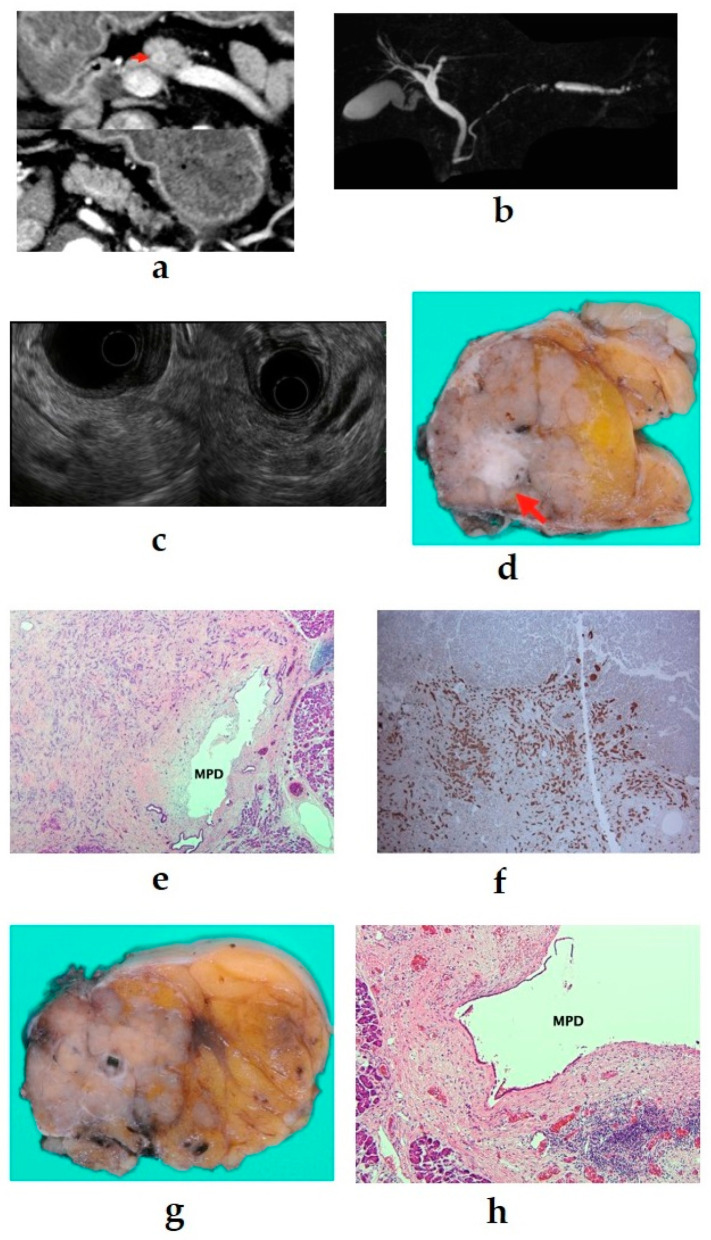
(**a**) Contrast-enhanced computed tomography (CT): a mass lesion with early hyperenhancement is seen in the pancreatic body (red arrow), but there are no other obvious lesions. (**b**) Magnetic resonance imaging: the main pancreatic duct (MPD) in the pancreatic body is extensively narrowed, and the caudal duct is dilated. (**c**) Endoscopic ultrasound (EUS): a circular hypoechoic mass in the pancreatic body. Pancreatic duct stenosis is observed even in the absence of mass. (**d**–**f**) 6-mm neuroendocrine neoplasm G1 (red arrow), serotonin positive, with stromal fibrosis (40×). (**g**,**h**) Pancreatic duct stenosis due to stromal fibrosis upstream of the tumor (40×).

Large PNENs may press the main pancreatic duct (MPD), resulting in stricture or obstruction; however, even small PNENs may cause MPD stenosis. It has been reported that pancreatic duct stenosis is caused not by physical compression by the tumor but by serotonin-induced stromal fibrosis [35,36]. Massironi et al. reported that CE-EUS does not show typical contrast-enhancement, that elastography shows a rigid pattern of the lesion, and that it is difficult to differentiate it from a pancreatic adenocarcinoma or an intraductal papillary mucinous neoplasm [37]. Examples of images of PNEN with MPD stenosis from our department are presented in Figure 4.

### 8.3. Intraductal Invasion of the MPD

Intraductal growth of PNENs is rare. It has been reported that tumors with intraductal growth are highly malignant and have a poor prognosis [38]. Intraductal invasion shows early contrast enhancement, with a decrease during the delayed phase on CT images, and this pattern helps differentiate pancreatic PNENs from pancreatic ductal adenocarcinomas. Under EUS, if the tumor extends to the MPD, malignancy must be considered. Acinar cell carcinomas and solid pseudopapillary neoplasms (SPNs) may also show intraductal growth, and these tumors may be difficult to differentiate from PNENs.

## 9. Features of Imaging Findings in PNEN G3 and Pancreatic Neuroendocrine Carcinoma (PNEC)

**Figure 5:**
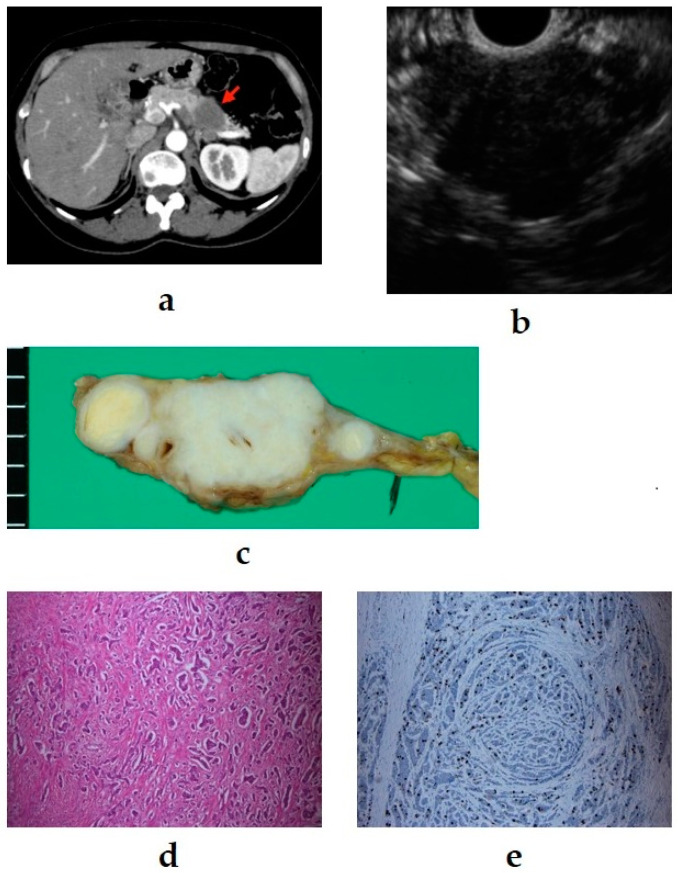
(**a**) Contrast-enhanced computed tomography (CT): an ill-defined mass (arrow) with a hypovascular enhancement pattern. The dilated pancreatic duct is notable. (**b**) Endoscopic ultrasound (EUS): irregular margins, unclear boundaries, and heterogeneous hypoechoic masses are shown. (**c**–**e**) Resected specimen: Ki-67 > 20%, neuroendocrine neoplasm G3 (100×).

Although there are histopathological differences between PNEN G3 and PNEC, their imaging findings are similar, and both of these tumors show similarities to normal pancreatic cancer (pancreatic ductal carcinoma) and pancreatic acinic cell carcinoma [39]. It has been reported that tumor blood flow correlates with prognostic factors, and the lower the vascularity, the more advanced the malignancy [40]. Histologically, the tumor does not have a capsule and grows invasively. Moreover, the tumor has abundant fibrous stroma, resulting in hypovascularity. The tumor margins are irregular, unclear, hypovascular, and there is internal necrosis of the tumor, and the above-mentioned pancreatic duct stenosis and intraductal extension occur at a high frequency. The necrotic area is recognized as a nonechoic area on B-mode EUS and as an avascular area on CE-EUS. It is difficult to distinguish using diagnostic imaging alone, and pathological examination is required. Examples of PNEN G3 images from our department are shown in Figure 5.

## 10. Tumors That Need to Be Differentiated from PNENs

### 10.1. SPNs

**Figure 6:**
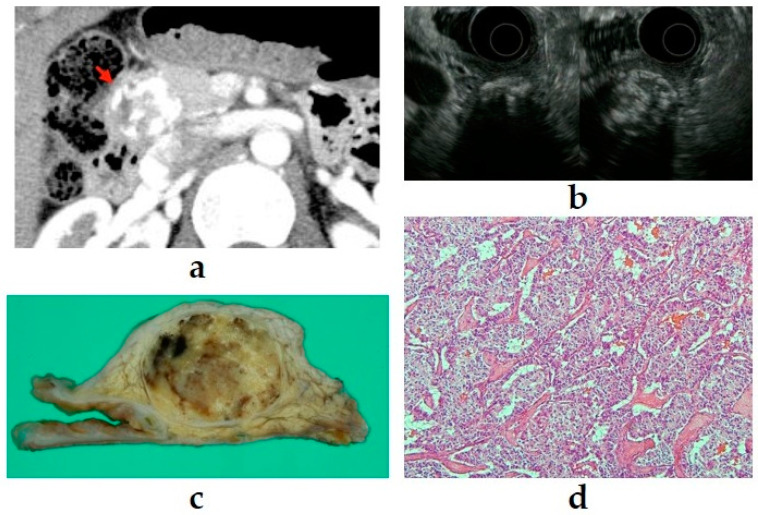
(**a**) Contrast-enhanced computed tomography (CT): a tumor (arrow) with calcification components is shown at the pancreatic head. (**b**) Endoscopic ultrasound (EUS): a well-defined mass with a heterogeneous appearance and peripheral rim calcification with posterior acoustic shadowing. (**c**,**d**) The imaging findings suggest a solid pseudopapillary neoplasm, but pathologically, it is a neuroendocrine tumor G1 (100×).

SPNs are mostly seen in young female patients, and most SPNs have a good prognosis [41]. SPNs usually show characteristics similar to those of PNENs, such as solid lesions with a round shape and clear borders within the pancreas, cystic degeneration, and cystic calcification. A study comparing the EUS findings of SPNs and PNENs reported that more SPNs had a cystic component and more PNENs had hypervascularity [42]. However, differentiation is often difficult, and pathological diagnosis with immunostaining using EUS-FNA is useful for diagnosis [43,44]. Examples of images of PNENs, similar to SPNs from our department, are presented in Figure 6.

### 10.2. Serous Cystic Neoplasm (SCN)

As mentioned above, cystic, degenerated PNENs show imaging findings similar to those of macrocystic-type SCNs. In addition, solid-type SCNs show imaging findings of hypervascularity and a solid appearance and need to be differentiated from typical PNENs [34,45]. Nonenhanced CT and MRI with T2-weighted imaging and apparent diffusion coefficient maps could be helpful for the differentiation because the cystic area of PNENs shows bleeding, whereas SCNs are different in that they store serous fluid. However, these tumors are difficult to distinguish using EUS alone.

### 10.3. Intrapancreatic Accessory Spleen (IPAS)

IPAS is a congenital ectopic spleen that mostly occurs in the pancreatic tail. IPAS appears as a well-defined circular mass and is hypervascular with a blood flow similar to that of the spleen [46,47]. Although it is difficult to distinguish IPAS from PNEN with EUS alone, Bhutani et al. reported that careful observation shows a bridge sign connecting the lesion and the spleen [48], and Ge et al. reported that EUS-elastography is useful for distinguishing it from PNEN [49]. In T2-weighted images of superparamagnetic iron oxide MRI, IPAS has a low signal similar to that of the spleen, thus allowing it to be distinguished from PNENs. The usefulness of histological diagnosis with EUS-FNA when differentiation is difficult with diagnostic imaging has been reported [50].

### 10.4. Pancreatic Metastasis

**Figure 7:**
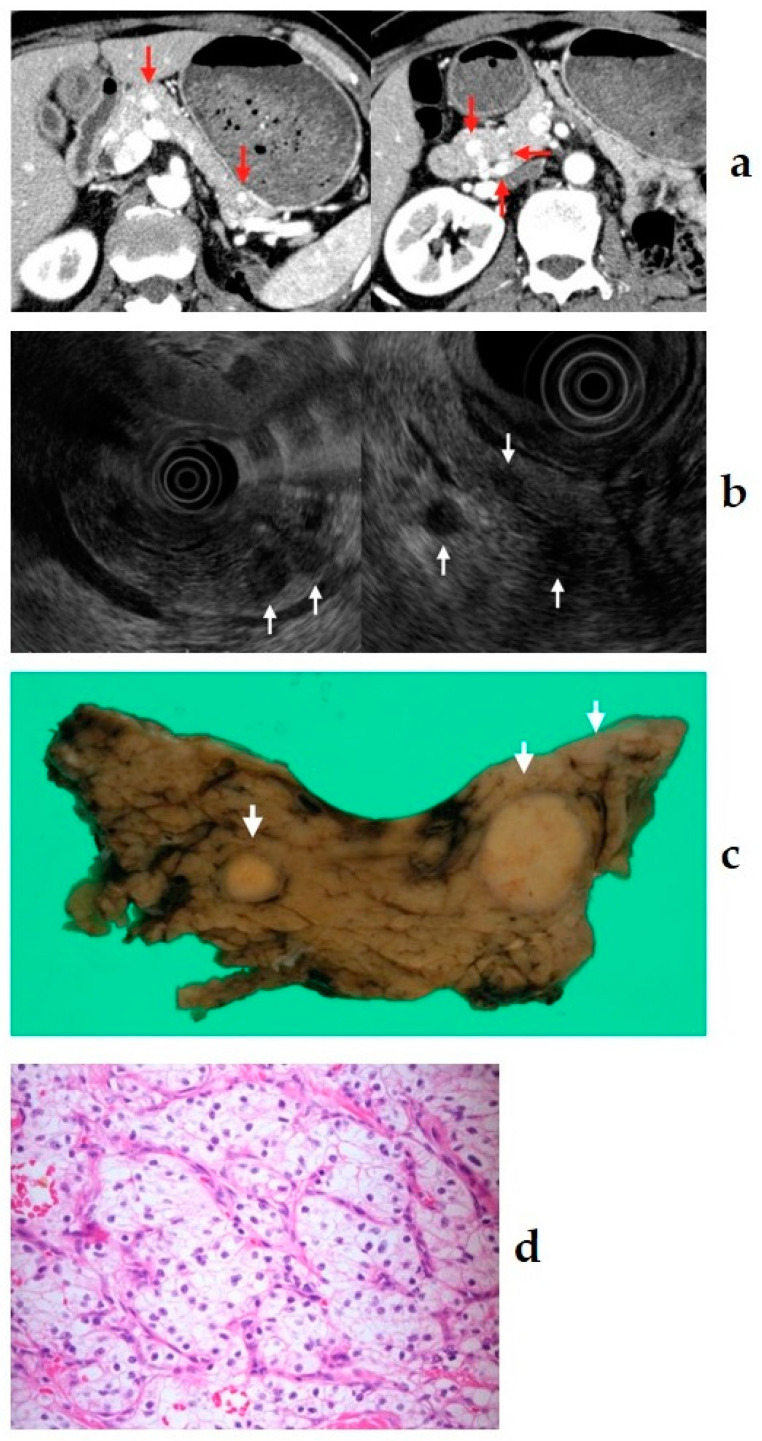
(**a**) Contrast-enhanced computed tomography (CT): many lesions with early hyperenhancement are seen in the head, body, and tail of the pancreas (red arrow). (**b**) Endoscopic ultrasound (EUS): many small hypoechoic masses are observed (white arrow). (**c**,**d**) Resected specimen: multiple tumors are observed throughout the pancreas (white arrow); they were clear cell carcinomas (400×). The patient had undergone left radical nephrectomy for renal cell carcinoma eight years ago.

Metastatic pancreatic tumors are relatively rare, and their imaging findings vary depending on the primary lesion. Renal cell carcinoma is the most common primary tumor of pancreatic metastasis. Metastatic pancreatic tumors from renal cell carcinoma become hypervascular tumors and show imaging findings similar to those of PNENs. Although information on the history of renal cell carcinoma is useful, these tumors are difficult to distinguish using imaging alone, and pathological histological diagnosis is required [51]. Examples of images of metastatic pancreatic tumors of renal cell carcinoma from our department are presented in Figure 7.

## 11. Role of EUS-FNA in PNENs

Tissue diagnosis and malignancy diagnosis using EUS-FNA are crucial for PNENs. Typical neuroendocrine tumors (NETs) need to be differentiated from SCNs, SPNs, and hypervascular pancreatic metastases. Atypical NETs, G3, and neuroendocrine carcinomas (NECs) need to be differentiated from normal pancreatic cancer and acinar cell carcinoma. These discriminations are difficult with diagnostic imaging alone, and tissue diagnosis plays a crucial role. The sensitivity and specificity of EUS-FNA for the diagnosis of PNENs are reported to be 73.2–100% and 83.3–93%, respectively [34,35,36,37,52,53,54,55]. Hijioka et al. reported that the location of the tumor in the pancreatic head and the presence of rich stromal fibrosis negatively affect the sampling adequacy of EUS-FNA [56]. In recent years, fine-needle biopsy (FNB) needles have been used, which are expected to improve diagnostic ability [57,58,59,60,61,62,63].

### 11.1. Grading Diagnosis

The grading of PNENs according to the World Health Organization (WHO) pathological classification (G1, G2, G3, and NEC according to the mitotic index and Ki-67 index) is essential for determining the treatment strategy. NEC in the WHO 2010 classification has been subdivided into G3 and NEC according to the WHO 2017 classification [64]. The treatment differed greatly between G3 and NEC.

In recent years, studies have shown that low-grade neuroendocrine neoplasms with a small diameter can be followed up without surgery [65,66]. As described above, grading diagnosis is important for the development of appropriate treatment strategies. The grading diagnosis requires a Ki-67 labeling index (LI) of the mitotic index. Mitoses were counted in 50 high-power fields (HPFs, 0.2 mm^2^) in areas of higher density and expressed as numbers per 10 HPFs (2.0 mm^2^). However, it is almost impossible to count in 50 HPFs with FNA samples, and the Ki-67 proliferation index is usually used in clinical practice. The concordance rate of the Ki-67 index between PNENs measured from EUS-FNA samples and surgical specimens was reported to be 54–100% [55,61,62,63,67,68,69,70,71,72,73,74,75,76,77,78,79,80,81,82,83,84,85] (Table 2), whereas a previous systematic review reported a rate of 83% [86]. Since the details of the data were unknown in a previous systematic review, we conducted a study by pooling the data of the studies that compared the Ki-67 LI grades obtained both in EUS-FNA samples and in surgical specimens. An extensive bibliographical search was performed in PubMed with the following search terms: “pancreas,” “pancreatic,” “neuroendocrine,” “NET,” “NEN,” “Ki-67,” “EUS,” and “endoscopic ultrasound” from January 2008 (according to the past review, the first report that studied cytological and surgical specimen was published in 2008) to October 2020. Additionally, the references of the selected studies and review articles were manually searched. The search was limited to human studies written in English. 

We extracted 25 articles comparing the Ki-67 LI grades obtained both in EUS-FNA samples and in surgical specimens [55,61,62,63,67,68,69,70,71,72,73,74,75,76,77,78,79,80,81,82,83,84,85,87,88]. Among them, Boutsen’s report was an additional study of Weynand’s report; therefore, Weynand’s report was excluded [71,79]. The number of articles that were graded according to the WHO 2010 or 2017 classification and whose detailed classification could be confirmed, or the articles in which the Ki-67 value was displayed and could be reclassified by us, was 22. Stratified analysis for tumors ≤ 2 cm was also performed; of the 22 articles, 10 reports showed the individual size or details of the classification ≤ 2 cm. Pooling the data of the studies, the concordance rate was 77.5% and the kappa correlation index was 0.65 (95% confidence interval (CI) = 0.59–0.71, *p* < 0.01) (Table 3) [55,61,62,63,67,68,69,70,72,73,74,75,76,77,78,79,80,81,82,83,84,87]. The sensitivity of G1 was good at 91.4% (338/370), but it was poor in G2 and G3 at 55.7% and 59.5%, respectively. The cause of this discrepancy was identified as intratumoral heterogeneity of Ki-67, and hot spots (areas with the highest fraction of positive tumor cells) were not observed. It is recommended to count more than 2000 cells to improve the grading diagnosis by EUS-FNA [89], and the WHO recommends counting more than 500 cells from hot spots [64]. It has also been reported that increased tumor size may contribute to increased intratumoral heterogeneity [74,76]. Stratified analysis of small tumor sizes showed that the concordance rate was 84.5% and the kappa correlation index was 0.59 (95% CI = 0.43–0.74, *p* < 0.01) (Table 4) [55,61,62,67,69,73,75,76,77,81,84]. The concordance rate of < 2 cm is higher, which is also proof that the larger the tumor size, the higher the intratumoral heterogeneity. On the contrary, a recent study reported that tumor differentiation and Ki-67 could be determined by EUS-FNA in only 26.4% and 20.1% of cases, respectively [55]. It is difficult to obtain enough tissue from a small tumor, which may be resolved using FNB needles.

### 11.2. EUS-FNA for Cystic PNENs

There are limited reports of EUS-FNA for cystic PNENs. The target of the puncture in cystic PNENs is a solid or cystic component. The cyst fluid was thin and clear, with low carcinoembryonic antigen (CEA) and amylase levels. Dhaliwal reported that the sensitivity of EUS-FNA for cystic P-NENs was 62.5%, which required FNA of both the solid and cystic components [18]. The cyst wall and septations should be targeted with FNA to maximize cytologic diagnosis [19]. In recent years, the usefulness of EUS-guided needle-based confocal laser endomicroscopy (nCLE) offering real-time microscopic imaging of the cyst epithelium providing virtual biopsies with high resolution (1–3.5 μm) has been reported [90,91]. It has also been reported to be useful in PNENs [92]. In addition, the efficacy of EUS-guided through-the-needle forceps biopsy (TTNB) for pancreatic cystic lesions has been reported [93,94,95,96]. The micro forceps, which is 0.8 mm in diameter, facilitates easy passage through a 19-gauge EUS-FNA needle and has a jaw-opening width of 4.3 mm, allowing for direct pancreatic cyst wall biopsy sampling. It is also possible to measure ki-67 in the TTNB specimens [97]. A recent study found that the combination of cyst fluid chemistry and cytology along with TTNB and/or nCLE results in a significantly higher diagnostic yield in pancreatic cystic lesions than any singular modality, although this was not statistically significant [98]. However, no large multicenter studies have specialized in cystic PNENs, even though these are necessary.

### 11.3. Genetic Analysis in PNENs

There are increasing reports on the use of next-generation sequencing with EUS-FNA samples in pancreatic tumors [99,100]. Recent studies have reported that alternative lengthening of telomeres, which are described as prognostic markers for resected PNENs, can be accurately performed on FNA specimens [101,102]. Genetic analysis of PNENs is also progressing [103]. A study with PNENs less than 3 cm identified genomic patterns of small PNENs associated with a different risk for liver metastases [104]. Cejas et al. elaborated on non-functional PNENs, which can predict the disease’s course and can give information on postoperative clinical decisions where enhancer maps that infer gene regulatory programs were used to classify the nonfunctional PNENs [105]. Young et al. performed a comprehensive analysis of the immune response and showed that immunotherapy may be clinically beneficial for patients with the metastasislike primary (MLP)-1 subtype [106]. In addition, Simon et al. performed multi-omics on PNENs of various grades and revealed the mechanisms involved in PNENs [107]. If PNENs can be further genetically analyzed and subdivided into Ki-67 grading before surgery, it will become an attractive option for the management and preoperative risk stratification of patients with PNENs.

## 12. Conclusions

The evolution of ultrasound imaging technologies such as contrast-enhanced and elastography and the AI that analyzes them, the evolution of FNB needles, and genetic analysis will further develop the diagnosis and treatment of PNENs in the future.

## Figures and Tables

**Table 1:** Imaging findings and differential diseases of pancreatic neuroendocrine neoplasms (PNENs).

	Findings	Differential Disease
PNENs (G1, G2)	Well-rounded, hypoechoic lesions with a homogeneous pattern and clear regular margins	SCN (solid type), SPN, metastic tumor, IPAS
	Cystic degeneration	SCN (macrocystic type), SPN
	Calcification	SPN
PNENs (G3, NEC)	Unclear irregular margins, hypovascular, and internal necrosis of the tumor	Pancreatic adenocarcinoma, acinar cell carcinoma
	Intraductal invasion of the main pancreatic duct	Acinar cell carcinoma

PNEN, pancreatic neuroendocrine neoplasm; SCN, Serious cystic neoplasm; SPN, solid pseudopapillary neoplasm; IPAS, Intrapancreatic Accessory Spleen; NEC, Neuroendocrine Carcinoma.

**Table 2:** Previous reports showing the concordance rates between EUS- fine-needle aspiration biopsy specimens and surgical specimens.

First Author	Year	Study Design	Number of Patients Analyzed for the Concordance Rate, *n*	Ki-67 Concordance Rate	Mean Lesion Size (Range), mm	Percentage of Functioning Tumor	Needle
Piani C [67]	2008	Retrospective	18	78–89% ^a^	30 (10–100)	38.9%	22-, or 25-gauge EUS-FNA needles
Kaklamatos M [68]	2011	Retrospective	26	54%	n.r.	n.r.	n.r.
Larghi A [69]	2012	Prospective	12	83.3%	16.9 (7–100)	0%	19-gauge EUS-FNA needles
Hasegawa T [70]	2014	Retrospective	27	77.8%	28.1 (5–130)	10.3%	25-, or 22-gauge EUS-FNA needles
Weynand B [71]	2014	Retrospective	33	57.6%	33 (2–110)	n.r.	22-gauge EUS-FNA needles
Carlinfante G [72]	2014	Retrospective	53	86.8%	17 (n.r.)	n.r.	25-, 19-, or 22-gauge EUS-FNA or EUS-FNB needles
Farrell JM [73]	2014	Retrospective	22	86%	30 (15–82)	24%	25-, 22-, or 19-gauge needles (details unknown)
Unno J [74]	2014	Retrospective	19	89.5%	22.3 (7–100)	31.6%	22-gauge EUS-FNA needles
Sugimoto M [75]	2015	Retrospective	8	87.5%	25.7 (4.4–10)	n.r.	25-, 22-, or 19-gauge EUS-FNA needles
Fujimori N [76]	2016	Retrospective	13	69.2%	20.5 (8–67)	13.1%	25-, or 22-gauge EUS-FNA needles
Díaz Del Arco C [77]	2016	Retrospective	10	70%	32 (12–120)	20%	n.r.
Laskiewicz L [78]	2018	Retrospective	26	84.6%	21 (8–140)	n.r.	n.r.
Boutsen L [79]	2018	Retrospective	57	72%	28.5 (2–110)	18.9%	n.r.
Weiss VL [80]	2018	Retrospective	49	61%	30 (n.r.)	6.1%	n.r.
Hwang HS [61]	2018	Retrospective	33	75.8%	33 (n.r.)	0%	25-, 22-, or 19-gauge EUS-FNB needles
Grosse C [81]	2019	Retrospective	15	100%	39 (9–75)	0%	n.r.
Di Leo M [62]	2019	Retrospective	25	84%	21 (n.r.)	n.r.	25- or 22-gauge EUS-FNA or 25-gauge EUS-FNB needles
Cui Y [82]	2020	Retrospective	37	73%	40 (7–170)	0	25-, 22-, or 19-gauge needles (details unknown)
Heidsma CM [55]	2020	Retrospective	63	81%	13 (n.r.)	14%	NA
Kalantri S [83]	2020	Retrospective	6 ^b^	100% ^b^	n.r. (11–70)	n.r.	22-gauge needles (details unknown)
Paiella S [84]	2020	Prospective	77	81.8%	24.5 (n.r.)	11.8%	25-gauge EUS-FNA needle
Kamata K [63]	2020	Prospective	23	82.6%	12.8 (n.r.)	n.r.	25-gauge EUS-FNB needle

^a^: Cut-off values of 2% and 89%. Cut-off values of 2%, 10%, and 78%. ^b^: 11 cases including non-surgical biopsy specimens were reported, with a concordance rate of 91%. n.r.: not reported. FNA, fine-needle aspiration; FNB, fine-needle biopsy.

**Table 3:** Concordance of PNEN grading between EUS-FNAB specimens and surgical specimens in pooling data [55,61,62,63,67,68,69,70,72,73,74,75,76,77,78,79,80,81,82,83,84,87].

	Resected Tumor Grade			
EUS-FNAB Tumor Grade	Grade 1	Grade 2	Grade 3	Total
Grade 1	338	88	5	431
Grade 2	32	111	12	155
Grade 3	0	0	23	23
Total	370	199	40	609

**Table 4:** Concordance of PNEN grading between EUS-FNAB specimens and surgical specimens in pooling data with small tumor sizes (2 cm or less) [55,61,62,67,69,73,75,76,77,81,84].

	Resected Tumor Grade			
EUS-FNAB Tumor Grade	Grade 1	Grade 2	Grade 3	Total
Grade 1	102	15	0	117
Grade 2	7	23	1	31
Grade 3	0	0	0	0
Total	109	38	1	148
